# DCVax®- DIRECT: autologous activated dendritic cells for image guided intra-tumoral vaccination in patients with solid tumors - a phase I/II clinical trial in progress

**DOI:** 10.1186/2051-1426-1-S1-P238

**Published:** 2013-11-07

**Authors:** Vivek Subbiah, Ravi Murthy, Chitra Hosing, Indresh Kaur, Gerald Falchook, Marnix Bosch

**Affiliations:** 1 Interventional Radiology, MD Anderson Cancer Center, Houston, TX, USA; 2 Investigational Cancer Therapuetics, Division of Cancer Medicine, MD Anderson Cancer Center, Houston, TX, USA; 3 Department of Stem Cell Therapy, Division of Cancer Medicine, MD Anderson Cancer Center, Houston, TX, USA; 4 Northwest Biotherapeutics, Inc., Bethesda, MD, USA

## 

Dendritic cells (DC) are acknowledged to be quintessential in the armamentarium to mount anti-tumor immune responses and have been utilized in varying capacities for cancer immunotherapy. Recent advancements & lessons leant from prior DC therapies have revealed that major barriers hinder the efficacy of cancer vaccination with DC, principal of which is the hostile environment of the local tumor milieu that inhibits activation and subsequent maturation of DC. This critical step is required to process and present antigens (tumor cell) to the downstream cascade of immune mediators. The therapeutic goals of cancer vaccination are the induction of tumor regression secondary to the production of tumor specific immune factors and local inflammatory cytokines with enhancement of long term anti-tumor surveillance to prevent recurrences. DCVax®- Direct (Northwest Biotherapeutics, Inc. Bethesda, MD) are autologous dendritic cells activated *Ex vivo* with BCG and IFNγ for intratumoral injection and attempts to circumvent this barrier thereby maximize the induction of anti-tumor responses. Autologous DC will be harvested from peripheral blood monocytes via leukapheresis. Following *Ex vivo* DC maturation, inoculation of the tumors will be performed 2 weeks later utilizing image guidance to ensure activated DC deposition at the peripheral aspect of the tumor thereby enhancing DC exposure to antigens from dead or dying tumor cells. Vaccination will be performed at least every week for 3 weeks, and subsequently at longer intervals dependent on harvested DC availability. Phase I/II study with DCVax®- Direct will enable evaluation of the safety, MTD, and responses in patients with solid tumors. The secondary objective addresses the feasibility, anti-tumor immune responses, PFS and OS. At the time of this poster submission, the 'First-in-man' patient has been consented for the study. We propose to present our initial findings at the SITC 2013 conference as more data will be available.

